# Sleep Habits in Pairs of Japanese High School Students and Their Mothers in Summer and Autumn

**DOI:** 10.3390/clockssleep4040041

**Published:** 2022-09-28

**Authors:** Koh Mizuno, Kazue Okamoto-Mizuno, Akiko Maeda

**Affiliations:** 1 Faculty of Education, Tohoku Fukushi University, Sendai 981-8522, Japan; 2 Faculty of Human Ecology, Wayo Women’s University, Ichikawa 272-0827, Japan; 3 Kansei Fukushi Research Center, Tohoku Fukushi University, Sendai 981-8522, Japan; 4 Faculty of Home Economics, Kyoritsu Women’s University, Tokyo 101-8437, Japan

**Keywords:** adolescents, mothers, seasonal variations, sleep environment

## Abstract

This study aimed to examine the sleep habits in pairs of Japanese high school students and their mothers in the summer and autumn. Nineteen pairs of high school students and their mothers participated in this study. Wrist actigraphy, subjective sleep evaluations, and bedroom environmental measurements (temperature, humidity, and light) were performed for a duration of one week. The results of a split-plot analysis of variance revealed no significant difference in the actigraphically evaluated time spent in bed (TIB) between the seasons and between the mothers and students. The TIB was approximately 6 h on weekdays, and significantly lengthened to approximately 7 h on weekends (*p* < 0.05). The average sleep efficiency values recorded were higher than 90%. The mothers showed significantly advanced sleep phases compared to those of the students (*p* < 0.05). In addition, the waking time on Monday morning was significantly correlated between the mothers and students in the summer and autumn (*p* < 0.05). A perceived sleep loss “almost every day” or “several times per week” was reported by approximately half of the mothers and students in each season. The students occasionally fell into nocturnal sleep with the room light turned on. These results suggest that sleep hygiene education considering life habit characteristics is required to ensure sufficient sleep time.

## 1. Introduction

Insufficient sleep is associated with adverse health and behavioral effects [1,2], and recently, the importance of adequate sleep on health has been widely discussed [3]. Since sleep habits and patterns are affected by demographic and social factors, such as age, sex, family, work, school, and technology, a considerable number of people experience insufficient sleep due to these factors [1]. From the viewpoint of an international comparison, Japan and Korea have the shortest sleep times worldwide. According to the results of time-use surveys conducted by the Organization for Economic Co-operation and Development (OECD) [4], the countries with the two shortest ranks of sleep time among the 33 countries (30 OECD countries and China, India, and South Africa) were Japan (442 min) and Korea (472 min), whereas the average sleep time for all the countries was 508 min (age range, 15–64 years). Among the results of the sleep time by the country and sex, Japanese women had the shortest sleep time of 435 min.

Based on the results of a domestic survey in Japan to determine the age- and sex-related characteristics, a nationwide survey conducted by the Ministry of Health, Labor and Welfare demonstrated that women in their 40s and 50s showed the shortest sleep time, revealing that approximately half of the respondents had a sleep time of less than 6 h [5]. The majority of these women might be housewives living with their spouse and children, struggling with housework to care for their family members, resulting in their sleep time being sacrificed. In addition, as these groups include the period of menopausal transition, when women often experience insomnia symptoms and increased sleep fragmentation [6], their short sleep may be insomniac and fragmented.

Although the survey by the OECD and the domestic survey in Japan did not cover the age groups below 15 and 20 years, respectively, the sleep time in Japanese adolescents is shorter than that in other countries. Currently, as sleep curtailment in adolescents is a worldwide issue, the sleep times in Japanese and Korean adolescents have been reported to be 1–2 h shorter than those in America, Australia, and the European countries [7,8]. A nationwide survey of Japanese junior and senior high school students (age range, 12–18 years) showed that 33.7% (boys, 31.4%; girls, 35.8%) of the respondents reported a sleep time on weekdays of less than 6 h, and that the sleep time became shorter with a higher school grade [9]. In addition, 57.7% of the boys and 64.5% of the girls engaged in daytime napping on weekdays. These napping behaviors increased with the increasing school grade, suggesting that the shorter sleep time in the higher school grades might be a cause of daytime napping.

Considering that the real life of a family consists of parents and children, these habits are mutually affected. With regard to adolescents and their parents, Fuligni et al. [10] reported a daily concordance between parent and adolescent sleep habits by analyzing the recordings of a two-week sleep log. The results of multilevel modeling demonstrated that the bedtime, waking time, and sleep duration are correlated daily between the adolescents and their primary caregivers. Further, such concordance was confirmed by the results of one-week actigraphic recordings as an objective sleep/wake evaluation [11]. Interestingly, this study showed a daily concordance in sleep habits between the children and their mothers but not between the children and their fathers. The association in sleep habits between children and their mothers has been reported in other studies using questionnaires, [12] and polysomnography and questionnaires [13].

Regarding the previous studies on the pairs of parents and their children, a study examining the influence of the seasons on sleep habits has not been conducted yet. Seasonal changes in sleep are well-documented. Typical cases [14,15] were reported in high-latitude areas (latitude, north [68–69°]), where the populations experience remarkable seasonal changes in daylight. In these areas, a phase-delayed nocturnal sleep period and a slight but significant decrease in sleep efficiency were more induced in winter compared to those in summer. Although those studies examined subjects living under real-life conditions by recording sleep using sleep logs or actigraphs, such as phase-delayed, the sleep in winter was also confirmed in Japan (latitude, north [43°]) using polysomnography within a controlled experimental facility [16]. As this study did not observe a decrease in sleep efficiency in winter, the seasonality of nocturnal sleep in Japan is characterized by an increase in awakenings in summer owing to hot and humid bedroom conditions. Studies examining Japanese community-dwelling elderly showed high bedroom temperatures and relative humidity, approximately 27 °C and 74%, respectively, accompanied by increased nocturnal awakenings in summer [17,18]. Therefore, summer may be the most severe season for Japanese adolescents and their mothers.

Taking the issues and facts described above into consideration, the present study examined the real-life situations of sleep habits in pairs of Japanese high school students and their mothers. To examine the seasonal differences in sleep habits, repeated measurements were conducted in the summer and autumn.

## 2. Results

### 2.1. Actigraphy

Table 1 presents the results of the actigraphic measurements. Regarding the sleep phase represented by bedtime and waking time, both revealed a significant delay in the high school students compared to their mothers (bedtime: F [1, 36] = 5.72, *p* < 0.05; waking time: F [1, 36] = 13.12, *p* < 0.001). In addition, waking times showed significant effects of the season (F [1, 36] = 4.31, *p* < 0.05) and day (F [2, 72] = 3.61, *p* < 0.05) and a significant interaction of the season × day (F [2, 72] = 24.24, *p* < 0.001). In both the mothers and high school students, and in both seasons, a significant delay in the weekend waking time compared to the weekdays was detected by post hoc comparisons. Further, the weekdays’ waking time in the high school students was significantly earlier in autumn than in summer. The weekend-delayed waking time resulted in a significant increase in the time in bed (TIB) on weekends (F [2, 72] = 6.16, *p* < 0.05). In addition, the TIB showed a significant interaction of the season × day (F [1.55, 55.91] = 7.44, *p* < 0.01), where the post hoc comparisons revealed that a significant increase in the weekends’ TIB in the high school students was detected in autumn, but not in summer. There was no significant difference in the TIB between the mothers and high school students. The total sleep time (TST) showed similar tendencies to those of the TIB, which showed no significant difference between the mothers and high school students, and a significant effect of the day (F [2, 72] = 5.80, *p* < 0.01), and a significant interaction of the season × day (F [1.50, 54.05] = 8.06, *p* < 0.01). A significant effect of the group was detected in the total waking time (TWT) (F [1, 36] = 13.12, *p* < 0.001) and the sleep efficiency index (SEI) (F [1, 36] = 4.98, *p* < 0.05). The high school students were characterized as having a longer TWT and lower SEI compared to the mothers. There was no significant effect of the group and season on social jet lag (SJL), revealing that the average values of sleep phase delay on weekends were approximately 30 min. In addition, no significant effect was detected in sleep latency (SL) and the number of wake episodes ≥ 5 min (NWE). With regard to the TST during the daytime in the mothers, a significant effect of the day was detected (F [1, 18] = 5.58, *p* < 0.05). In both summer and autumn, the TST during the daytime was approximately 20 min longer on weekends than on weekdays.

Table 2 presents the correlations of each actigraphic sleep parameter between the mothers and the high school students. The parameter to demonstrate a statistically significant correlation both in summer and autumn was the waking time at the beginning of the week. The other parameters to show significant positive correlations were the TIB at the beginning of the week and the TWT and SEI on the weekdays in summer.

### 2.2. Bedroom Environment

Table 3 presents the results of the environmental measurements in the participants’ bedrooms. A significant effect of the season was detected on the average bedroom temperature (F [1, 36] = 162, *p* < 0.0001) and relative humidity (F [1, 36] = 146, *p* < 0.0001). Although the bedroom temperature in autumn was optimal (22 ± 3 °C), the bedroom temperature in summer (27 ± 2 °C) was considered close to the upper limit of the temperature required for sufficient sleep. The average bedroom temperature was significantly correlated between the mothers and the high school students, both in summer (r = 0.89, *p* < 0.0000001) and autumn (r = 0.81, *p* < 0.00001). However, the average bedroom relative humidity was significantly correlated only in summer (r = 0.89, *p* < 0.0000001) but not in autumn (r = 0.46, *p* = 0.05). As for bedroom light levels during the TIB, a significant effect of the group was detected both in the weekly averaged values of the number of nights when illumination ≥ 200 lx was recorded (F [1, 36] = 9.74, *p* < 0.01) and the duration of the illumination ≥ 200 lx (F [1, 36] = 6.52, *p* < 0.05). These results indicate that the occasions that the room light remained turned on during the TIB was more frequent in the high school students than in the mothers.

### 2.3. Questionnaires

Table 4 shows the results of the sleep health questionnaire (SHQ) answered by the mothers and the high school students. There were no significant seasonal effects on any of the factor scores. Both the mothers and the high school students showed high average scores of approximately 65 in the factor score of sleep initiation. For the other four factor scores, significant differences between the mothers and high school students were detected. The sleep maintenance scores (F [1, 36] = 6.23, *p* < 0.05) and respiration scores (F [1, 36] = 8.47, *p* < 0.01) were higher in the high school students than in the mothers, and the parasomnia scores (F [1, 36] = 23.42, *p* < 0.001) and waking-up scores (F [1, 36] = 6.18, *p* < 0.05) were higher in the mothers than in the high school students.

Table 5 shows the domestic behavioral characteristics that might affect sleep. Among these parameters, no significant seasonal effects were detected. The time spent watching TV was significantly longer on weekends than on weekdays (F [1, 36] = 64.29, *p* < 0.00001), and a significant interaction of the day × group × season (F [1, 36] = 7.92, *p* < 0.01) was detected. The results of the post hoc comparison showed that the time spent watching TV on weekends in autumn was significantly longer in the mothers than in the high school students. In the high school students, the time spent studying was significantly longer on weekends than on weekdays (F [1, 18] = 17.01, *p* < 0.001). The time spent using electronic devices showed a significant effect of the day (F [1, 36] = 4.19, *p* < 0.05; longer on weekends than on weekdays), the group (F [1, 36] = 4.90, *p* < 0.05; longer in the high school students than in the mothers), and a significant interaction of the day × group (F [1, 36] = 9.79, *p* < 0.01). The results of the post hoc analysis indicated that the time spent using electronic devices on weekends by the high school students was significantly longer than that on weekdays and weekends by the mothers. The frequency of using electronic devices within 30 min before bedtime showed no statistically significant seasonal and group differences. The percentage of the participants who answered “always” ranged between 58% and 74%.

Table 6 presents the daytime sleep issues, which were the frequencies of perceiving sleep loss, dozing off and/or taking a nap, and napping after returning home in the high school students. Based on the result of the χ^2^ test, there was no significant effect of the season or group. The percentage of the participants who perceived sleep loss “almost every day” ranged from 21% to 37%. As for the factors that cause sleep loss, the results of the χ^2^ test revealed no significant effect of the season on sleep loss in the mothers and the high school students. The most frequent answer in the high school students was “homework given by the high school”, which was answered by 76% and 73% of the high school students in summer and autumn, respectively. The second most frequent answer was “using electronic devices such as a smartphone”, which was answered by 53% and 33% of the high school students in summer and autumn, respectively. The other major factor was “extracurricular club activities” in autumn, which was answered by 33% of the high school students. In summer, “extracurricular club activities” was answered by 24% of the high school students. With regard to the mothers, the most frequent answers were family-related issues and domestic affairs, which were answered by 50% and 44% of the mothers in summer, respectively. Although these percentages increased to 78% and 61% in autumn, respectively, the results of the χ^2^ test revealed no significant effect of the season on the sleep loss in autumn (*p* = 0.08 and 0.38). The percentage of the mothers who answered other causes was below 30%.

### 2.4. Sex-Specific Differences in Sleep among the High School Students

There was no significant difference in any of the actigraphic sleep parameters between the female and male high school students. Regarding the results of the questionnaires, the males (summer: 58 ± 5; autumn: 58 ± 4) scored significantly higher in the waking up item of the SHQ than the females (summer: 53 ± 9; autumn: 51 ± 7) (F [1, 17] = 5.41, *p* < 0.05). In addition, the females perceived sleep loss in autumn significantly more frequently than the males (*p* < 0.05). Although half of the females responded that they perceived sleep loss “almost every day”, 88% (8 of 9) of the males answered that they “never” or “rarely” perceived sleep loss. Regarding the factors that cause sleep loss, “extracurricular activities” in autumn was listed by 5 of 10 females, which is higher (*p* = 0.053) than the number of males who reported the same response (0 out of 5). There was no significant sex-specific difference in the other factors shown in Supplemental S1. 

## 3. Discussion

The present study aimed to examine the real-life situations of the sleep habits in pairs of Japanese high school students and their mothers, who are known to experience the shortest sleep worldwide. From the viewpoint of the seasonal influences, one of the repeated measurements was conducted in summer, which is the most severe season due to hot and humid bedroom conditions. The results of the actigraphic measurements indicated no significant seasonal differences in the TIB and TST. Approximately 6 h of the TIB with a high SEI above 90% was recorded in both the mothers and high school students in both seasons. Compared with the results of previous nationwide epidemiological studies on Japanese women [5] and adolescents [9], which showed that a considerable proportion of the population had a sleep time of less than 6 h, the TIB in the present study was consistent with those studies. Their short sleep time might induce a high sleep propensity, resulting in a high SEI, even in summer. Although the previous studies [17,18] examining Japanese elderly people have reported that a summer bedroom temperature similar to the present study is accompanied by an increase in nocturnal awakenings; the mothers and high school students may be able to sleep under such hot and humid bedroom conditions since they are younger than the elderly. However, since approximately half of the participants answered that they perceived sleep loss “almost every day” or “several times per week” (Table 6), it is evident that their sleep time was insufficient.

Significant differences in the bedtime and waking time were detected, indicating that the sleep phase in the mothers was advanced compared with that in the high school students. Since Japanese wives engage in most of the housework [19,20], the earlier waking time in the mothers might be due to the morning housework, such as cooking breakfast and preparing lunch boxes. In addition, the waking time showed significant effects of the day and season, and a significant interaction between the season and day. The waking time was significantly more delayed on the weekends, compared to the weekdays, and advanced in autumn compared to summer, and the weekday waking time in the high school students was significantly earlier in autumn than in summer. These differences were considered to be due to social factors, rather than seasonal environmental changes, namely, the condition depending on the high school activities. As the data collection period in autumn was conducted during the school term, the significantly earlier waking time on weekdays in the high school students was for attending regular high school classes, which began at 8:30 a.m. In summer, although the high school students went to school to attend supplemental classes and/or extracurricular club activities 3–5 days/week, it was confirmed that those activities began later than the regular classes during the school term. Interestingly, the bedtime did not show such weekly and seasonal influences. Some of the participants went to bed earlier on weekends than on weekdays. Although the reasons for no significant change in bedtime were unclear, this might be due to chronic sleep loss, which appeared as a possible measure to ensure or make sleep time. As a result of the regular weekly bedtime, a mean SJL of approximately 30 min was identified to be included in a group of the mildest or no SJL reported in the previous studies on Japanese adults [21] and adolescents [22]. SJL has been associated with various adverse effects, such as impairments in work and academic performance, aggression and conduct problems, and cardiometabolic risks [23]. Although the present participants showed almost no SJL, they might experience adverse effects associated with chronic sleep loss. The absence of SJL may largely be due to school activities on weekends, such as club activities that start in the morning. It could be difficult to compensate for sleep loss by delaying waking time, and the only way to compensate for this might be going to bed early. This might explain why some of the high school students went to bed earlier on the weekends. Future studies should compare the health and behavioral conditions between the groups with and without SJL but under chronic sleep loss, that is, those who could not settle the weekdays’ sleep debt on the weekends.

The association or concordance in nocturnal sleep between mothers and children has been confirmed in the previous studies [10,11,13]. Recently, Kouros and El-Sheikh [11] reported a significant association between children and their mothers in actigraphically evaluated TST, TWT, SEI, and waking time. They analyzed the results of 163 pairs using multilevel models to demonstrate a daily concordance in the recordings of one-week actigraphy. In addition, a significant correlation in the results of single-night polysomnography between adolescents and their mothers has been reported [13]. By performing home-based polysomnography on 47 families, a significant correlation was detected in the TST, SL, SEI, light sleep, and slow-wave sleep. Unfortunately, multilevel modeling could not be applied in the present study because of the small sample size. The present results of correlations in the sleep parameters (Table 3) were partially consistent with those of the previous studies that showed a significant correlation in some parameters on weekdays and at the beginning of the week. Interestingly, no significant correlation was found between the parameters on the weekends. These results suggest that the association of sleep habits between the mothers and the high school students weakens on weekends because of decreased social and familial constraints. In addition, the significant positive correlations between the mothers and the high school students in the TIB on the beginning of the week, and the TWT and SEI on weekdays, were detected only in summer. From the viewpoint of the bedroom thermal conditions, the bedroom temperature was correlated between the mothers and the high school students. Meanwhile, the average bedroom temperature ranged between 25 to 30 °C and 18 to 25 °C in summer and autumn, respectively. These results suggest that a hot bedroom environment in summer is a factor that induces a correlation or concordance in nocturnal sleep between mothers and children. 

Nationwide studies on Japanese adolescents have demonstrated that females are at a higher risk of having self-reported sleep problems, such as a short sleep duration, subjective insufficient sleep, and excessive daytime sleepiness, than males [9,24]. Although there were no sex-specific differences in actigraphy sleep parameters in the present study, the results showed that the females had significantly lower waking up scores in the SHQ and a higher frequency of perceiving sleep loss in the autumn than the males. Although the number of participants in the present study is small, the results indicate that females may be prone to more severe self-reported sleep problems than males, even under similar objective sleep conditions. Another sex-specific difference noted in this study was the difference in the cause of sleep loss in autumn. Five females and no males reported that their loss of sleep was caused by “extracurricular activities”. Three out of the five females were in a brass band club. Meanwhile, no students out of the other 10 respondents, who did not report “extracurricular activities” as a cause of sleep loss, were in a brass band club. Thus, the brass band club activities may have characteristics, such as lengthy practice times, that could cause sleep loss.

The results of the SHQ revealed that both the mothers and the high school students were at approximately the same or higher than the average of Japanese adults. Among the five factor scores, the highest values were for the sleep initiation score, which was found in both the mothers and the high school students. This might be ascribed to the increased sleep propensity due to the short sleep time. The significantly lower values of the sleep maintenance and respiration scores observed in the mothers were considered to be age-related influences. The decrements in sleep maintenance [25] and increases in respiration problems during sleep [26] are known to increase with age. Parasomnia symptoms, such as sleep paralysis, are more frequent in adolescents [27], and adolescents’ sleep phase tends to be delayed [28]. These could be the reasons for the significantly low parasomnia and waking up scores in the high school students compared to those in the mothers. In addition, as there was no difference in the TIB and TST between the mothers and the high school students, the low waking up scores in the high school students might be partially induced by a more severe sleep loss compared to that in the mothers. The National Sleep Foundation’s sleep duration recommendations [29] propose that the sufficient sleep times for the general population are 8–10 h for teenagers (14–17 years) and 7–9 h for adults (24–64 years), and less than 7 and 6 h are not recommended for teenagers and adults, respectively. Therefore, the sleep time in both the mothers and the high school students in the present study (approximately 6 h) was short, and the high school students might experience more severe sleep loss because they require more sleep than the mothers. It was interesting to note that the high school students seemed to have the occasion that the room light remained turned on during the TIB (Table 4). A possible case for this situation was that the high school students fell asleep without turning the room lights off due to a strong sleep propensity. This could occur under the condition of severe sleep loss, but such nocturnal sleep must be of low quality with intermittent awakenings, and possibly a decreased melatonin secretion owing to the bright environment [30]. Therefore, although higher sleep maintenance scores were observed in the high school students than in the mothers, the actual sleep conditions might be worse in the high school students.

This study has some limitations. First, the high school students did not wear the actigraphs while at school. Therefore, although the responses in the questionnaire showed that approximately 40% of the high school students frequently or sometimes took naps or dozed off during the day, the possible daytime naps during school hours were not objectively monitored. These results suggest that naps or dozing off while in school or during a commute may partially compensate for the sleep loss in the high school students. However, Lo et al. [31] demonstrated that the neurobehavioral functions of adolescents in a condition of restricted nocturnal sleep (TIB, 5 h), with a 1 h daytime nap, is lower than those recorded in a condition of sufficient nocturnal sleep (TIB, 9 h) without daytime naps. Further, Santos et al. [32] reported that high school students with napping habits show increased daytime sleepiness and poor nocturnal sleep quality. Therefore, if the students took naps or dozed off while they were not wearing the actigraphs, the possible beneficial effects of the naps may be insufficient, and/or the students may experience increased daytime sleepiness and poor sleep quality. 

Second, as suggested [33], the sleep onset identified by the actigraphy might occur before the polysomnographically evaluated stage-1 sleep. Therefore, some student cases that indicate the room lights to be on during the TIB might include polysomnographically awake periods. However, among the 65 nights (in 10 and 9 students in summer and autumn, respectively) recording an illumination of 200 lx or higher during the TIB, the number of nights with this high illumination during the TIB for more than 30, 60, and 120 min were 22, 10, and 6 nights, respectively. Thus, as the review paper mentioned above [33] indicated that long, continuous periods of sleep are scored as sleep by both polysomnography and actigraphy, it was considered that they slept without turning the room lights off on some nights, revealing long sleep episodes under a high environmental light. Third, the objective sleep evaluation in this study is by one-week wrist actigraphy. A longer measurement period, such as two weeks, may show more informative and valid results, especially for the data on weekends, because only one weekend of data could be affected by some accidental reason.

The results of the previous nationwide studies on sleep problems in adults [34] and adolescents [9,24] conducted in Japan indicate the need for health education as a solution for sleep problems. In agreement with the findings of those studies, the present results in the mothers and high school students suggests that sleep hygiene education is needed for both groups. Regarding the behavioral aspects presented in Table 6 and Table 7, watching TV seemed to have no adverse influence on sleep in the present participants. The use of electronic devices such as smartphones has been suggested to be associated with sleep problems in adolescents [35,36]. A notable finding was that the time spent using electronic devices by the high school students was remarkably higher on weekends compared to weekdays and that in the mothers. Since more than 30% of the high school students listed electronic device use as a factor causing sleep loss, appropriate education should be recommended, especially for high school students. The other point to be taught to high school students should be to avoid napping after returning home, which was answered by 25% of the students, who take a nap “several times a week” or “almost every day”. Although taking a short nap in the early afternoon, which is around the timing of the circadian dip, is known to have benefits such as preventing sleepiness and improving cognitive functions in the afternoon after the nap [37], napping after returning home in the late afternoon to evening has been reported to be associated with a later bedtime and daytime sleep impairments, such as excessive sleepiness [36,38]. The percentage in this study was relatively lower than that reported in the previous studies of Japanese adolescents [36,38].

With regard to the mothers, possible measures to save sufficient sleep time might be to reduce the family-related issues and domestic affairs, which were answered by approximately 50% or more of the mothers as a cause of sleep loss. For this purpose, it is essential to share the idea of “fair housework-sharing among the family”. Kobayashi et al. [20] reported that sharing housework is associated with marital satisfaction. From the viewpoint of saving sufficient sleep time, this issue is not only for marital satisfaction but also for various other issues, including health, work and academic performance, and familial management.

In conclusion, the present study examined the real-life situations of the sleep habits in pairs of Japanese high school students and their mothers. The results of repeated measures in the summer and autumn showed no seasonal differences in the subjective and objective sleep parameters except for an earlier waking time in the autumn. The waking time on Monday was significantly correlated between the groups in both seasons. Both groups showed short sleep times, and approximately half of the participants in each group perceived sleep loss “almost every day” or “several times per week”. Compared to the mothers, the high school students, especially the females, showed significantly lower scores in the subjectively evaluated waking up state, and they occasionally fell into nocturnal sleep with the room light turned on. These findings suggest that high school students might be under the condition of severer sleep loss compared to that of the mothers. As has been suggested by the results of national surveys [9,24,34], sleep hygiene education considering people’s life habit characteristics and familial situations is required to ensure sufficient sleep time. 

## 4. Materials and Methods

### 4.1. Participants and Procedure

Pairs of high school students and their mothers were recruited from Sendai City, Japan (latitude, north [38°]). As for the high school students, we selected the 10th and 11th grades (from 15 to 17 years) because the students in the highest grade (12th) might have begun devoting themselves to the university entrance examination, resulting in significant lifestyle changes, such as shortening the nocturnal sleep time and refraining from club activities. The other inclusion criteria for the pairs were as follows: participants who were healthy and not receiving medical treatment, participants without insomnia, participants who were nonsmokers, and participants not taking care of other family members, such as grandparents or children aged younger than two years. Initially, 20 pairs participated in the measurements, and 19 pairs completed the full sets of measurements. The means and standard deviations of the physical characteristics of the high school students and their mothers are shown in Table 7. Among the 19 high school students, 12 (7 boys and 5 girls) and 6 students (1 boy and 5 girls) belonged to athletic and cultural clubs, respectively. However, one male student did not belong to any of the clubs in high school and went to a private tutoring school after his high school classes five days per week. Among the 19 mothers, 14 were working four or more days per week; 3 worked occasionally or two or three days per week; and 2 did not work.

Repeated measurements of the pairs were conducted in the summer and the following autumn. The measurements consisted of one-week wrist actigraphy to evaluate the sleep/wake rhythm, continuous measurements of bedroom environmental conditions (ambient temperature, humidity and light levels), and use of a paper-based questionnaire, which is shown in Supplemental S1. The questionnaire and a set of devices were handed to the participants. For the female high school students and the mothers, the measurements were not conducted during menstruation. The measurement schedule was planned based on the menstrual cycle, which was conducted by female staff. As a result of this schedule adjustment, the periods for summer and autumn measurements were from 5 July to 31 August and 25 September to 3 November, respectively. Although the 1-month summer vacation of high school began in late July, the high school students went to high school to attend supplemental classes and/or extracurricular club activities three to five days/week. The measurements in summer were conducted on 10, 7 and 2 students during summer vacation, school term, and the periods across the summer vacation and school term, respectively.

### 4.2. Measures

#### 4.2.1. Actigraphy

One-week wrist actigraphy was performed using a Micro Motion Logger (Ambulatory Monitoring Inc., New York, NY, USA). The participants were requested to wear an actigraph on the wrist of their nondominant hand continuously, except while bathing. For the high school students, to prevent the loss and destruction of the actigraph, the actigraph was not worn when at school. The participants were requested to keep a written sleep log and to track their bedtime, waking time and the time when the actigraph was temporarily removed. Th actigraphic recordings were analyzed with commercial software (Action-W, 2.4.20, Ambulatory Monitoring Inc., New York, NY, USA) using the Cole–Kripke algorithm for scoring sleeping/waking [39]. Over the course of the recordings, the bedtime, waking time, and TIB (defined as the primary sleep period during which participants were trying to or falling into sleep) were determined nightly by the suggestions of the software and the participants’ sleep logs. The TIB, SL (time from bedtime to sleep onset), TWT, TST, NWE and SEI (percentage of sleeping time scored in TIB) were calculated using the software. For these sleep parameters, the mean values of the weekdays (Monday night to Thursday night) and weekends (Friday and Saturday night) were calculated. The Sunday night data were categorized as “the beginning of week”. As the mothers continuously wore the actigraph, the TST during the daytime (from waking time to bedtime) was calculated, and the mean values of the weekdays (from Monday to Friday) and weekends (Saturday and Sunday) were calculated. Finally, the relative SJL was determined by the results of the one-week actigraphy, calculating the difference between the mean values of the midpoint of the sleep period time on the weekends and weekdays. The sleep period was determined from the sleep onset to the last sleep episode in the TIB. 

#### 4.2.2. Bedroom Environmental Measurements

During the actigraphy measurement period, the ambient temperature, relative humidity and light levels in the participants’ bedrooms were recorded every 5 min using a TR 74Ui (T and D Corp, Matsumoto, Japan). The participants were instructed to place the device adjacent to their heads while they slept. For the ambient temperature and relative humidity, the average values during the individual’s TIB were calculated. Then, the weekly average values were calculated based on the nightly average values for assessing the sleep thermal condition in each of the summer and autumn data collections.

As for the results of the light levels, illumination of 200 lx or more during the TIB was considered to be the case when the participants slept under bright conditions. The number of nights when illumination ≥ 200 lx was recorded during the TIB was counted among the seven recording nights. Furthermore, the duration of recording illumination ≥ 200 lx during the TIB each night was estimated. Because the light levels were recorded every 5 min, consecutive episodes with illumination ≥ 200 lx were converted to the estimated duration by multiplying the number of episodes by 5 min. Regarding a single recorded episode, although the actual duration, which might be less than 1 min and up to nearly 10 min, was unclear, it was converted to 2.5 min. The total duration over the one-week measurement period was divided by 7 to calculate the nightly average value.

#### 4.2.3. Questionnaires

In the period of the second half of the actigraphy measurement, the participants were requested to answer a set of questionnaires asking about their life and sleep habits, looking back over the previous month. A detailed outline of the questionnaire, which consists of items on the (1) SHQ; (2) time spent watching TV, studying (only for the high school students), and using electronic devices (such as a smartphone); (3) perception of sleep loss and factors that cause sleep loss; (4) and frequency of dozing off and/or taking naps, is shown in the Supplemental S1. The SHQ contains 14 questions, which are questions 1 to 14 in Supplemental S1. The results were used to calculate the T-scores for five sleep health factors: sleep maintenance, parasomnia, respiration, sleep initiation and rising in the morning. These T-scores are based on a database of Japanese adults, and the higher the score, the better the sleep health [40]. The other items are not standardized; however, they were used to investigate the life and sleep habits in our previous study on Japanese high school students [36]. Questions on the time spent watching TV, studying, and using electronic devices, such as smartphones or electronic games, on weekdays and weekends were included in the questionnaire (questions 15 to 17). In addition, the frequency of using electronic devices within the 30 min before bedtime was assessed (question 18). Regarding the question on the perception of sleep loss (question 19), a follow-up question (question 20) on the factors that cause sleep loss was included in the questionnaire. Based on the results of the preliminary interviews, several possible causes of sleep loss were provided as possible responses to the question. Regarding the question on the frequency of dozing off and/or taking naps (question 21), the answer categories were the same as those in the SHQ (question 4 to 11), because this question is a supplementary question included in the Japanese version of the SHQ. For the other questions on the frequency of behaviors or sensations (questions 18, 19, and 22), the answer categories were partially different from those for question number 21. For the high school students, napping behavior after returning home was investigated (question 22) because this behavior has been reported to be associated with daytime sleep impairments, such as excessive sleepiness [36,38]. 

### 4.3. Statistical Analyses

To examine the statistical significance, we used a split-plot analysis of variance for the continuous numerical data, where the between-participants factor was the group (the high school students and their mothers), and its within-participants factors consisted of the season (summer and autumn) and day (weekdays, weekends, and the beginning of week, or weekdays and holidays). If Mauchly’s sphericity test was significant (*p* < 0.05), the level of significance was considered after a Greenhouse–Geisser correction for the repeated measures. When the results revealed significant interactions or a significant effect of the day (weekdays, weekends, and the beginning of week), a post hoc analysis was performed using Tukey’s test for the between-participant comparison and a paired t-test with a Bonferroni correction for the within-participant comparison. The correlations of each actigraphic sleep parameter and the average bedroom temperature between the mothers and the high school students were examined using Pearson’s r. As for the categorical data asking the frequency of the participants’ behaviors, such as dozing off and/or taking a nap, the χ^2^ test was used. To examine the sex-specific differences in the sleep characteristics of the high school students, a split-plot ANOVA, with the season being the within-participant factor, was utilized for the analysis of the SHQ scores and actigraphy sleep parameters. The sex-specific differences in the factors that cause sleep loss and the frequencies of perceiving sleep loss, dozing off and/or taking a nap, and napping after returning home were examined using the χ^2^ test. The statistical analyses were performed using PASW Statistics 17.0.2 (IBM Corporation, Armonk, NY, USA). The level of significance was set at *p* < 0.05.

## Figures and Tables

**Table 1:** The results of actigraphic measurements.

	Summer						Autumn					
	Weekdays		Weekends		Sunday Night		Weekdays		Weekends		Sunday Night	
	Mothers	Students	Mothers	Students	Mothers	Students	Mothers	Students	Mothers	Students	Mothers	Students
Bed time ^(^^a)^	23:33 ± 0:14	24:30 ± 0:14	23:52 ± 0:13	24:43 ± 0:22	23:30 ± 0:15	24:55 ± 0:17	23:37 ± 0:13	24:04 ± 0:14	23:35 ± 0:16	23:55 ± 0:21	23:48 ± 0:16	23:51 ± 0:31
Wake time ^(bcdg)^	5:35 ± 0:12	6:50 ± 0:15	6:31 ± 0:15 ^#^	7:44 ± 0:25 ^#^	6:00 ± 0:15 ^#^	7:15 ± 0:19	5:30 ± 0:12	6:11 ± 0:10 ^†^	6:22 ± 0:17 ^#^	7:31 ± 0:22 ^#$^	5:53 ± 0:14	6:27 ± 0:19
Duration (min) TIB ^(df)^	363 ± 12	381 ± 16	400 ± 14 ^#^	421 ± 16	391 ± 19	381 ± 21	354 ± 8	364 ± 14	407 ± 9 ^#$^	457 ± 27 ^#^	366 ± 13	397 ± 29
SL	6.8 ± 1.2	9.5 ± 1.1	6.4 ± 1.4	6.9 ± 0.7	6.4 ± 1.4	6.0 ± 1.2	8.6 ± 0.9	7.5 ± 1.0	8.3 ± 1.1	6.6 ± 1.5	8.9 ± 2.4	9.6 ± 1.8
TWT ^(b)^	18 ± 3	33 ± 7	22 ± 4	31 ± 5	18 ± 4	32 ± 5	16 ± 2	25 ± 4	19 ± 2	29 ± 6	19 ± 3	35 ± 5
TST ^(ef)^	344 ± 11	347 ± 15	378 ± 11 ^#^	390 ± 15	372 ± 20	349 ± 16	338 ± 8	339 ± 12	388 ± 9 ^#$^	428 ± 26 ^#^	346 ± 12	363 ± 27
NWE	1.3 ± 0.3	2.0 ± 0.4	1.2 ± 0.2	2.0 ± 0.4	1.7 ± 0.5	1.8 ± 0.4	1.2 ± 0.1	1.6 ± 0.3	1.3 ± 0.2	1.9 ± 0.4	1.4 ± 0.2	2.2 ± 0.3
SEI ^(a)^ (%)	95 ± 1	92 ± 2	95 ± 1	93 ± 1	95 ± 1	93 ± 1	95 ± 0	93 ± 1	95 ± 1	94 ± 1	95 ± 1	91 ± 1
Day TST ^(c)^ (min)	28 ± 17	-	47 ± 39	-	-	-	33 ± 25	-	50 ± 56	-	-	-
SJL (min)	Mothers			Students			Mothers			Students		
	37 ± 10			32 ± 15			26 ± 5			37 ± 14		

Results are shown as means ± standard deviation. ^(a)^ significant effect of group (*p* < 0.05); ^(b)^ significant effect of group (*p* < 0.001); ^(c)^ significant effect of season (*p* < 0.05); ^(d)^ significant effect of days (*p* < 0.05); ^(e)^ significant effect of days (*p* < 0.01); ^(f)^ significant interaction between season × day (*p* < 0.01); ^(g)^ significant interaction between season × day (*p* < 0.001). ^#^ significantly differed from weekdays; ^$^ significantly differed from Sunday night; ^†^ significantly differed from summer. TIB, time in bed; SL, sleep latency; TWT, total wake time; TST, total sleep time; NWE, the number of wake episodes ≥ 5 min; SEI, sleep efficiency index; Day TST, total sleep time during the day; SJL, social jet lag.

**Table 2:** Correlation coefficients in sleep parameters between the mothers and high school students.

		Bed Time	Waking Time	TIB	TWT	TST	SEI	SL	NWE	SJL
Summer	Weekdays	0.23	0.30	0.27	0.59 *	0.20	0.58*	0.10	0.42	0.17
	Weekends	0.00	−0.26	−0.10	−0.24	0.00	−0.23	−0.21	0.06	
	Beginning of the week	0.20	0.77 **	0.52 *	0.08	0.29	0.14	0.37	0.06	
Autumn	Weekdays	0.25	0.42	0.26	−0.03	0.15	−0.12	−0.17	0.07	−0.32
	Weekends	0.17	−0.25	0.42	0.00	0.28	−0.10	0.03	0.13	
	Beginning of the week	−0.04	0.54 *	0.20	−0.36	0.15	−0.42	−0.12	−0.43	

* *p* < 0.05; ** *p* < 0.01. TIB, time in bed; TWT, total wake time; TST, total sleep time; SEI, sleep efficiency index; SL, sleep latency; NWE, the number of wake episodes ≥ 5 min.

**Table 3:** The results of bedroom environmental conditions during TIB.

	Summer		Autumn	
	Mothers	Students	Mothers	Students
Temperature (°C) ^(a)^	27 ± 1	27 ± 2	22 ± 3	22 ± 3
Relative humidity (RH%) ^(a)^	63 ± 5	65 ± 5	59 ± 5	60 ± 6
The number of nights a light intensity of 200 lx or higher was recorded ^(c)^	0.0 ± 0.0	0.7 ± 1.2	0.2 ± 0.5	0.8 ± 1.4
The duration that an illumination of 200 lx or higher was recorded during TIB (min) ^(b)^	0.1 ± 0.3	7.7 ± 15.8	1.6 ± 4.0	14.8 ± 30.6

Results are shown as means ± standard deviation. ^(a)^ significant effect of season (*p* < 0.0001); ^(b)^ significant effect of group (*p* < 0.05); ^(c)^ significant effect of group (*p* < 0.01).

**Table 4:** The results of SHQ.

	Summer		Autumn	
	Mothers	Students	Mothers	Students
Sleep maintenance ^(a)^	57 ± 6	60 ± 5	58 ± 7	62 ± 3
Parasomnia ^(c)^	62 ± 1	57 ± 5	62 ± 2	57 ± 5
Respiration ^(b)^	49 ± 10	58 ± 7	51 ± 11	58 ± 8
Waking up ^(a)^	59 ± 4	55 ± 8	59 ± 5	54 ± 7
Sleep initiation	65 ± 5	63 ± 6	66 ± 5	64 ± 6

Results are shown as means ± standard deviation. ^(a)^ significant effect of group (*p* < 0.05); ^(b)^ significant effect of group (*p* < 0.01); ^(c)^ significant effect of group (*p* < 0.001).

**Table 5:** The results of domestic behavioral characteristics.

	Summer					Autumn				
	Weekdays		Weekends			Weekdays		Weekends		
(min)	Mothers	Students	Mothers	Students		Mothers	Students	Mothers		Students
Time spent Watching TV ^(bf)^	103 ± 97	56 ± 36	141 ± 107 ^#^	115 ± 63 ^#^		96 ± 97	53 ± 35	159 ± 110 ^#^^†^		98 ± 55 ^#^
Studying ^(a)^	-	68 ± 41	-	137 ± 109		-	72 ± 45	-		120 ± 87
Using electronic devices ^(cde)^	102 ± 126	126 ± 68	92 ± 51 ^†^	208 ± 114 ^#^		128 ± 144	114 ± 57	105 ± 67 ^†^		191 ± 84 ^#^
			**Summer**				**Autumn**			
	**(%/n)**		**Mothers**		**Students**		**Mothers**		**Students**	
Frequency of using electronic devices within 30 min before bedtime	Almost everyday		68/13		58/11		74/14		74/14	
	Several times/week		16/3		11/2		5/1		16/3	
	Rarely		11/2		16/3		11/2		5/1	
	Never		5/1		16/3		11/2		5/1	

Results are shown as means ± standard deviation. ^(a)^ significant effect of day (*p* < 0.001); ^(b)^ significant effect of day (*p* < 0.00001); ^(c)^ significant effect of day (*p* < 0.05); ^(d)^ significant effect of group (*p* < 0.05); ^(e)^ significant interaction between group × day (*p* < 0.01); ^(f)^ significant interaction between group × day × season (*p* < 0.01). ^#^ significantly differed from weekdays; ^†^ significantly differed from the high school students.

**Table 6:** The results of daytime sleep issues.

(%/n)		Summer		Autumn	
		Mothers	Students	Mothers	Students
Frequency of perceiving sleep loss	Almost everyday	21/4	37/7	32/6	32/6
	Several times/week	37/7	26/5	16/3	11/2
	Rarely	37/7	26/5	48/9	37/7
	Never	5/1	11/2	5/1	21/4
Frequency of dozing off and/or taking a nap	Frequently	5/1	16/3	26/5	5/1
	Sometimes	48/9	26/5	32/6	32/6
	Occasionally	32/6	37/7	21/4	37/7
	Never	16/3	21/4	21/4	26/5
Frequency of napping after returning home	Almost every day	-	5/1	-	21/4
	Several times/week	-	21/4	-	5/1
	Rarely	-	31/6	-	16/3
	Never	-	42/8	-	58/11

**Table 7:** Physical characteristics of the participants.

		High School Students	
	Mothers (n = 19)	Female (n = 10)	Male (n = 9)
Age (years)	48.2 ± 2.8	16.1 ± 0.6	15.9 ± 0.8
Height (cm)	158 ± 4.4	155 ± 4.2	171 ± 3.2
Weight (kg)	52 ± 6.1	48 ± 5.9	57 ± 6.4
BMI	21 ± 2.3	20 ± 1.9	20 ± 2.3
Belonging to athletic/cultural club	-	5/5	7/1
Working/not working	17/2	-	-

## Data Availability

Data sharing not applicable.

## Supplementary Materials

The following supporting information can be downloaded at: https://www.mdpi.com/article/10.3390/clockssleep4040041/s1, Supplemental S1. The Questionnaire.
