# Supplementary material for: Sleep Habits in Pairs of Japanese High School Students and Their Mothers in Summer and Autumn
Source: Clocks Sleep. 2022 Sep 28;4(4):520–34. doi: 10.3390/clockssleep4040041 (PMC9589961; doi:10.3390/clockssleep4040041)
Supplement: Supplementary file 1 [file clockssleep-04-00041-s001.zip › clockssleep-1886387-supplementary.pdf]

Supplemental S1. The Questionnaire

ID#:______ Date:______ Height:______cm Weight:______kg

The following questions ask your sleep and life habits. Please answer the questions in the second-half of the actigraphic measurement period. Please answer all the, and questions with looking back over the previous month.

1. How many times during the night (during sleep) do you wake up?
   (1) None (2) About___times per night

2. How deep is your usual sleep?
   (1) Sound (2) Generally sound (3) Neither (4) Generally poor (5) Poor

3. About how many times do you go to the toilet during the night?
   (1) Do not go (2) Go about___times per night

4. Do you awake too early in the morning?
   (1) Frequently (2) Sometimes (3) Occasionally (4) Never

5. Are you told you are groggy at night?
   (1) Frequently (2) Sometimes (3) Occasionally (4) Never

6. When sleeping at night are you struck with a feeling of being tied down (Kanashibari)?
   (1) Frequently (2) Sometimes (3) Occasionally (4) Never

7. Do you have vivid, frightening dreams as you are about to fall asleep at night?
   (1) Frequently (2) Sometimes (3) Occasionally (4) Never

8. Are you told your legs twitch or kick during the night, or do your legs become restless and uncomfortable when you get sleepy?
   (1) Frequently (2) Sometimes (3) Occasionally (4) Never

9. Do you take sleeping medicine or tranquilizers to sleep?
   (1) Frequently (2) Sometimes (3) Occasionally (4) Never

10. Do you snore?
   (1) Frequently (2) Sometimes (3) Occasionally (4) Never

11. Are you told that you stop breathing during your sleep?
   (1) Frequently (2) Sometimes (3) Occasionally (4) Never

12. Can you get out of bed when it is time to get up?
   (1) I can get up easily (2) I can get up with a little effort
   (3) I can get up with considerable effort (4) I cannot get up no matter how hard I try

13. How long does it normally take you to fall asleep after getting into bed?
Generally about___minutes

14. How long does it take you get up after waking in the morning?
Generally about___minutes

15. How long do you watch TV?
On weekdays: (1) None (2) About___minutes per day
On weekends: (1) None (2) About___minutes per day

16. How long do you study? (Only for the high school students)
On weekdays: (1) None (2) About___minutes per day
On weekends: (1) None (2) About___minutes per day

17. How long do you use electronic devices such as smartphones or electronic games?
On weekdays: (1) None (2) About___minutes per day
On weekends: (1) None (2) About___minutes per day

18. Do you use electronic devices within 30 min before bedtime?
(1) Almost every day (2) Several times/week (3) Rarely (4) Never

19. Do you perceive sleep loss?
(1) Almost every day (2) Several times/week (3) Rarely (4) Never

20. If you perceive sleep loss, check all cause(s) show below.
☐ Using electronic devices such as a smartphone;
☐ Watching TV;
☐ Insomnia or stress;
☐ No specific cause;
☐ Long commuting time to the workplace/school;
☐ Others;

Only for mothers:
☐ Domestic affairs;
☐ Family-related issues;
☐ Job-related issues;

Only for high school students:
☐ Homework given by the high school;
☐ Extracurricular activity

21. Do you doze off and/or take a nap?
(1) Frequently (2) Sometimes (3) Occasionally (4) Never

22. Do you take a nap after returning home from school? (Only for the high school students)
(1) Almost every day (2) Several times/week (3) Rarely (4) Never
